# Systemic Lymphadenopathy as the Initial Presentation of Malignant Mesothelioma: A Report of Three Cases

**DOI:** 10.4061/2010/846571

**Published:** 2010-01-04

**Authors:** Yaxia Zhang, Zohreh M. Taheri, Merce Jorda

**Affiliations:** Department of Pathology, University of Miami-Miller School of Medicine/Jackson Memorial Hospital, Miami, FL 33136, USA

## Abstract

Systemic lymph node metastasis is a rare event in malignant mesothelioma. It is even more exceptional when systemic lymph node metastasis is the initial clinical presentation. Review of literature discloses only four cases in which metastatic lymphadenopathy was the only symptom of malignant mesothelioma. We, herewith, report three cases where the initial diagnosis of malignant mesothelioma was made by biopsy of enlarged lymph nodes, which were the only clinical presentation. Immunohistochemistry played a pivotal role in elucidating the mesothelial origin of their unusual systemic lymph node metastasis.

## 1. Introduction

Malignant mesothelioma (MM) is an uncommon neoplasm which is characterized by highly aggressive behavior and poor prognosis [1]. The neoplasm predominantly involves pleural and peritoneal cavities, with a smaller percentage of cases arising in the pericardial sac and testicular tunica vaginalis [2]. Clinically, the majority of patients have local symptoms such as chest or abdominal pain and dyspnea, depending on the site of origin. Occasionally, patients may present with distant metastasis. Systemic lymphadenopathy, however, is an exceeding rare initial presentation of this disease [3]; thirteen cases have been reported in literature. In four of those, systemic lymphadenopathy was the only clinical manifestation [4–6]. 

 In this report, we describe three additional cases of primary peritoneal MM in which the initial diagnosis of the disease was made by biopsy of neck, supraclavicular lymph nodes, and axillary lymph nodes. Two cases were primary from peritoneum and the third originated in the pleura.

## 2. Report of Cases

### 2.1. Case One

**Figure 1:**
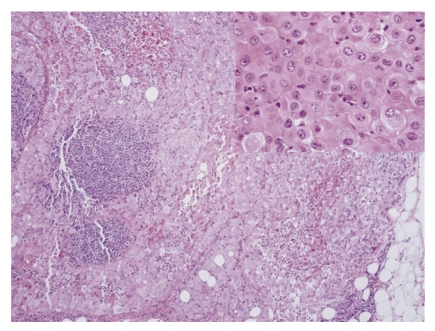
Metastatic malignant mesothelioma to lymph node. (H&E, 10×). The inset shows that the tumor cells are polygonal with well-defined cell membranes and dense eosinophilic cytoplasm (H&E, 60×).

**Figure 2:**
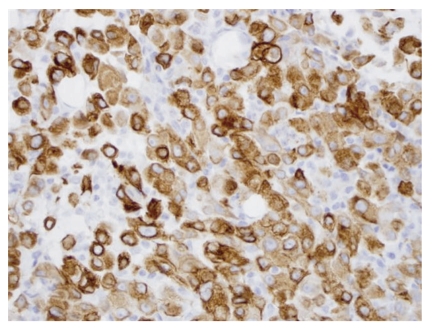
Metastatic malignant mesothelioma to lymph node. The tumor cells are positive for cytokeratin by immunohistochemistry (Keratin, 40×).

**Figure 3:**
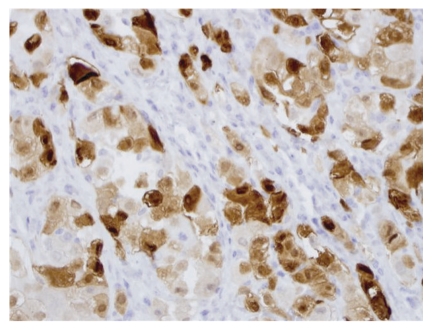
Metastatic malignant mesothelioma to lymph node. The tumor cells are positive for calretinin by immunohistochemistry (Calretinin, 40×).

**Figure 4:**
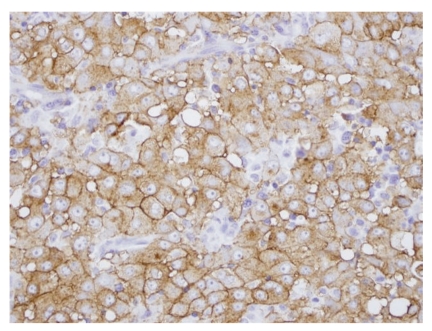
Metastatic malignant mesothelioma to lymph node. The tumor cells are positive for D2-40 by immunohistochemistry (D2-40, 40×).

A 50-year-old male with no past medical history presented with progressive enlargement of lymph nodes in his left groin and right inferior neck over a period of 6 months. PET scan showed high uptake in several areas such as mediastinum, pericardial region, cardiophrenic angles, supraclavicular area, internal mammary, perihepatic region, and groin with standardized uptake values (SUVs) ranging from 4.8 to 12.5. Most of these uptakes were interpreted as presence of a malignant process probably malignant lymphoma. Immediate assessment on fine needle aspiration cytology from left groin lymph node, however, ruled out the diagnosis of malignant lymphoma. A subsequent core biopsy was obtained from right supraclavicular node and was interpreted as metastatic malignant epithelial neoplasm to lymph node. The neoplasm was characterized by a diffuse growth of polygonal cells with well-defined cell membranes and dense eosinophilic cytoplasm. Nuclei were generally single, and mitoses were scant (Figure 1). Tumor cells were positive for cytokeratin (Figure 2), calretinin (Figure 3), D2-40 (Figure 4), CD10, and CK5/6, focally positive for CK7 and CA-125, and negative for hepatocellular antigen, renal carcinoma antigen, prostatic specific antigen, carcinoembryonic antigen, HMB45, S100 protein, p63, CDX2, CK20, thyroid transcription factor-1, inhibin, and alpha-feto protein by immunohistochemistry. Based on histomorpholgy and immunophenotype, the diagnosis of metastatic malignant mesothelioma was rendered. Retrospectively, review of clinical and imaging studies confirmed that the neoplasm arised from peritoneum in perihepatic region.

### 2.2. Case Two

**Figure 5:**
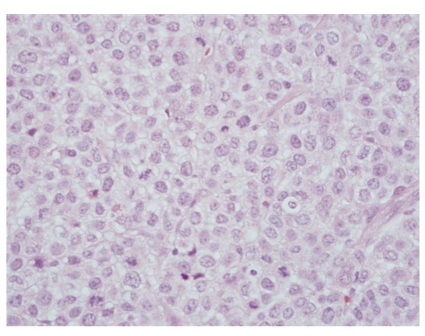
Metastatic malignant mesothelioma to lymph node. The tumor cells are large and polygonal with eosinophilic cytoplasm. They have prominent nucleoli and display numerous mitoses (H&E, 40×).

A 61-year-old male with no past medical history presented with left neck lymphadenopathy. CT scan showed multiple masses in the neck region, mediastinum and abdomen. Biopsy from neck mass revealed several matted lymph nodes with complete replacement by a nonlymphoid malignant neoplasm. The cells were arranged in organoid and trabecular pattern with areas of necrosis with perinodal tumoral involvement. The tumor cells were large and polygonal with eosinophilic cytoplasm, large prominent nucleoli, and numerous mitoses (Figure 5). Tumor cells were positive for cytokeratin and calretinin by immunohistochemistry. They were negative for CD20, CD45, CD3, S-100 protein, thyroglobulin, carcinoembryonic antigen, human chorionic gonadotropin, keratin 8/18, thyroid transcription factor-1, inhibin, and renal carcinoma antigen. A diagnosis of metastatic malignant mesothelioma was made. Retrospectively, review of prior CT scans revealed that the neoplasm originated in pelvic peritoneum.

### 2.3. Case Three

Patient is a 69-year-old male who by imaging proved to have mediastinal and left axillary lymphadenopathy. Biopsy of the axillary lymph node showed a metastatic epithelial neoplasm morphologically consistent with malignant mesothelioma. Positive immunohistochemical reaction for calretinin and negative staining for TTF-1 and CEA supported that diagnosis. Clinical and imaging studies revealed left pleural thickening. A pleural biopsy confirmed the diagnosis of epithelioid malignant mesothelioma.

## 3. Comment

Malignant mesothelioma is characterized by an aggressive local behavior, such as pain and accumulation of fluid in the region of origin [7]. Rarely, this neoplasm metastasizes to lymph nodes, particularly early in the course of disease. In the majority of cases, metastatic spread to the lymph nodes is detected simultaneously with other conventional clinical features of malignant mesothelioma, such as pain and pleural or peritoneal effusions. Malignant mesothelioma presenting with peripheral lymphadenopathy as the initial symptom is exceptionally rare. To our knowledge, there have been 13 cases of MM with clinically apparent and histopathologically proven lymph node metastasis [4–13] but in only 4 of them, metastatic lymphadenopathy was the initial clinical presentation. Two of these cases were originated in peritoneum [4], each from pleura [6], and pericardium [5]. In our case one, PET scan showed numerous systemic enlarged lymph nodes, with the largest lymph node present at the mediastinum. Retrospectively, the area with high standardized uptake values present at the perihepatic region was considered to be the primary site of the neoplasm. Similarly, in our case two, retrospective analysis of imaging studies revealed the site of origin. In our case three, the biopsy from pleural surface confirmed the diagnosis of MM which was initially made by sampling patient's axillary lymph node. 

 When one confronts a metastatic epithelial neoplasm to a lymph node with evidence of a primary site, a malignant mesothelioma is usually not on the list of potential differential diagnoses. A combination of histomorphology along with an appropriate immunohistochemical tests, however, may elucidate the mesothelial origin of such rare metastasis. Immunohistochemistry is the most helpful ancillary test in confirmation of the diagnosis, particularly in separating MM from adenocarcinoma. Most useful markers in our experience from MM are calretinin and D2-40 and negative reaction for CEA, MOC-31, and TTF-1 [14, 15].
